# Possible benefit of second intravenous immunoglobulin course in Guillain−Barré syndrome patients with potentially poor prognosis

**DOI:** 10.1111/ene.16231

**Published:** 2024-02-05

**Authors:** Hai‐Feng Li, Zuneng Lu

**Affiliations:** ^1^ Department of Neurology Xuanwu Hospital, Capital Medical University Beijing China; ^2^ Department of Neurology Renmin Hospital of Wuhan University Hubei China

## CONFLICT OF INTEREST STATEMENT

None.

Dear Editor,

In this comprehensive guideline [1], a strong recommendation was proposed against giving a second intravenous immunoglobulin (IVIg) course in Guillain−Barré syndrome (GBS) patients with a poor prognosis. This is exclusively based on the negative results and potential increase of thromboembolic events in the SID‐GBS trial [2]. Howevere, we have three concerns about this study.

- The modified Erasmus GBS Outcome Score (mEGOS) ≥6 points acquired 7–9 days after initiation of the standard course was used to recruit patients with poor prognosis for the second intravenous immunoglobulin dose (SID) [2]. In a study using the inability to walk independently at 6 months as the indicator of poor prognosis, the mEGOS score acquired on admission was lower compared with that acquired 7 days after admission but the variation of scores was relatively larger [3]. Using the mEGOS score on admission may recruit more patients with variable severity whose prognoses were potentially poor, some of whom would potentially benefit from SID in the early course. Moreover, patients with mEGOS ≥7 points on admission were found to benefit from intensive immunotherapy [3].
- Although the disability score at 4 weeks is the most frequently used outcome measure in the trials of GBS, its predictive significance in relation to long‐term prognosis has not been validated. The inability to walk independently at 6 months is more meaningful for the recovery of GBS. In the SID‐GBS trial, the scores at 4, 8 and 12 weeks were in skewed distribution, with the median close to the severe end. However, the scores at 26 weeks were close to a normal distribution and the score of the SID group was closer to the mild end [2]. This implied that some patients might benefit from SID.
- Since IVIg takes effect predominantly in the early course, the intervals between onset to the standard IVIg course is important. However, this information was not provided and compared amongst all groups (including SID, one standard course and placebo). One study found that days from weakness onset to admission were negatively associated with inability to walk unaided at 6 months [4], whilst a shorter interval between onset and admission was found to be associated with mechanical ventilation in another study [5].

Therefore, we would anticipate that the benefit of SID might be found with some modification of the study design, including selecting poor‐outcome patients based on the risk score on admission instead of after the standard course, taking the inability to walk independently at 6 months as the primary end‐point, and using the interval between onset and first course as a covariate for adjustment.

Furthermore, the SID‐GBS trial found that patients with thromboembolic events did not have higher immunoglobulin concentrations after one standard IVIg course or SID [2]. More severe disease in patients receiving SID might be related to more adverse events, including thromboembolic events.

Considering the devastating effect of poor GBS outcomes, we argue for a possible benefit of SID in patients with potentially poor prognosis, especially by giving SID immediately after the standard course when there is still progression.

## AUTHOR CONTRIBUTIONS

**Hai‐Feng Li:** Conceptualization; writing – original draft; writing – review and editing. **Zuneng Lu:** Writing – review and editing; writing – original draft.

## Data Availability

Data sharing is not applicable to this article as no new data were created or analyzed in this study.
